# Analytical factors for eight short-chain fatty acid analyses in mouse feces through headspace solid-phase microextraction–triple quadrupole gas chromatography tandem mass spectrometry

**DOI:** 10.1007/s00216-023-04895-1

**Published:** 2023-08-17

**Authors:** Sunhee Kang, Jeonghyun Yun, Ho-Young Park, Jang-Eun Lee

**Affiliations:** 1 Fermented Food Research Group, Food Convergence Research Division, Korea Food Research Institute, 245 Nongsaenmyeong-Ro, Wanju-Gun, Jeollabuk-Do 55365 Republic of Korea; 2 Food Functionality Research Division, Korea Food Research Institute, 245 Nongsaenmyeong-Ro, Wanju-Gun, Jeollabuk-Do 55365 Republic of Korea; 3 Department of Food Biotechnology, University of Science and Technology, Daejeon, 34113 Republic of Korea

**Keywords:** Short-chain fatty acid, Mouse feces, GC-MSMS, Solid-phase microextraction (SPME), Salting out

## Abstract

This study developed a method for quantifying eight short-chain fatty acids (SCFAs) in mouse fecal samples using solid-phase microextraction (SPME) coupled with triple quadrupole gas chromatography tandem mass spectrometry. Furthermore, significant factors affecting SCFA analysis, including SPME fiber selection, pH, salting-out agent, and sample collection time, were investigated. Contrary to previous studies, we found that the CAR/PDMS fiber had the highest extraction efficiency for all SCFAs. The optimal extraction efficiency was observed at pH 2.0, particularly for low-molecular-weight SCFAs. NaH_2_PO_4_ showed a more effective extraction efficiency than NaCl, owing to its pH stability and less interference with the solvent matrix. Additionally, our results showed that the SCFA concentration increased over collection time. The composition ratio of the eight SCFAs was maintained for up to 24 h; thus, we concluded that samples should be collected within four hours to obtain reliable results. Our findings may improve laboratory methods for SCFA extraction and mouse fecal sample analysis.

**Graphical Abstract:**

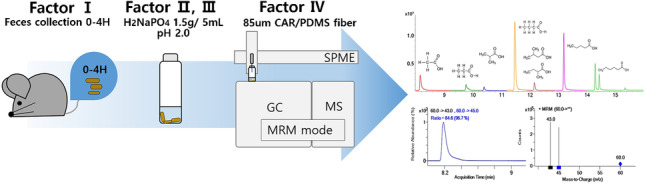

**Supplementary information:**

The online version contains supplementary material available at 10.1007/s00216-023-04895-1.

## Introduction

Short-chain fatty acids (SCFAs) are carboxylic organic acids with less than six carbon atoms that are mainly produced from the gut microbial fermentation of dietary fiber and non-digestible polysaccharides [1]. The most significant and abundant SCFAs are acetic (C2), propionic (C3), and butyric (C4) acids. Aside from these compounds, other C4–C6 SCFAs, such as valeric and hexanoic acids, have been extensively investigated in the gut microbiota for their potential health benefits.

Numerous studies have been conducted on the physiological roles of SCFAs in human health and body functions. In the past decade, there has been increasing interest in the analysis of SCFAs in diverse biological materials such as plasma [2, 3], serum [3, 4], mouse feces [5], human stools [6–8], urine [6], and fermented media [9]. In particular, according to recent literature, different sample preparation and analytical methods as well as the reliability of the results of SCFA analysis in human feces, have been systematically reviewed and summarized. However, numerous studies have used mouse models and have been trying to estimate SCFA levels in mouse feces. The fact that only 5% of all SCFAs produced by microbes can be found in the feces is not sufficient to explain the intestinal condition and gut microbiota [10]. Nevertheless, using fecal samples is considered noninvasive and universal, and a number of reliable results on the correlation between the health benefits and fecal content of SCFAs have been widely reported. Compared to human feces, the most significant factor that should be considered in SCFA analysis using mouse feces is that only an extremely small amount of sample can be obtained, which leads to the investigation of increasing the analytical efficiency of the SCFA analysis method. In SCFA analysis in mouse feces, some critical preparation and analytical steps should be considered to increase analytical efficiency, such as fecal sample collection, SCFA extraction, and instrumental conditions.

In addition, according to recent scientific literature, gas chromatography (GC) is the primary analytical technique for quantifying SCFAs due to its sensitivity and specificity in identifying and measuring individual SCFAs. In addition, alternative methods have also been reported in various studies, including liquid chromatography (LC), which can be particularly useful when analyzing complex matrices due to its excellent resolution, as well as nuclear magnetic resonance (NMR), which provides a non-destructive and highly reproducible method for the quantification of SCFAs, though it is less sensitive compared to chromatographic techniques. However, despite the availability of these alternatives, GC is the most widely used method for SCFA quantification, reflecting its robustness, accuracy, and reliability [11]. In the past, most researchers had used pretreatment procedures, such as derivatization, extraction, or distillation, but recently there has been a noticeable increase in the use of solid-phase microextraction (SPME) coupled with GC–MS in SCFA quantification studies. However, most studies analyzing SCFAs primarily use human feces or blood samples rather than mouse stool samples. Therefore, there is a need for more research aimed at overcoming the challenges associated with conducting these analyses using limited sample quantities.

In the present study, we aimed to develop a method for analyzing SCFAs in mouse fecal samples using headspace solid-phase microextraction (SPME) coupled with gas chromatography tandem mass spectrometry (GC–MS/MS). Among many analytical factors, we focus on extraction conditions under the SPME method, including the feces matrix effect depending on salt and pH, and investigated the effect of mouse feces freshness to confirm the sample collecting condition. The result contains the method development of eight kinds of SCFAs through the different SPME fiber extraction, pH and salting-out effect, triple quadrupole of GC–MS/MS condition, and reliability of results depending on feces sample freshness. Thus, in the present study, given the importance of SCFA analysis in mouse fecal samples, it aimed to develop a sensitive and reliable method to increase the headspace SPME extraction efficiency of SCFAs and suggest how the mouse fecal sample should be collected. Our results will help other researchers in the analysis of SCFAs not only in mouse fecal samples but also in other human and animal biological materials.

## Materials and methods

### Chemicals and reagents

Acetic acid (99.7%), acetic acid-1-_13_C (99%), propionic acid (99.5%), propionic acid-d_2_ (99%), isobutyric acid (99%), butyric acid (99.5%), butyric acid-1-_13_C (99%), 2-methylbutyric acid (97%), isovaleric acid (98.5%), valeric acid (99.8%), valeric acid-_13_C (99%), hexanoic acid (99%), hexanoic acid-d_11_ (99%), monosodium phosphate (NaH_2_PO_4_, 99%), sodium chloride (NaCl, 99.5%), and sulfuric acid (99.9%) were purchased from Sigma-Aldrich (St. Louis, MO, USA). Water (liquid chromatography–mass spectrometry grade) was obtained from Merck (Darmstadt, Germany).

### Stock solution and sample preparation

To prepare the standard mixed SCFA stock solution, acetic (1.00 g), propionic (0.10 g), isobutyric (0.05 g), butyric (0.05 g), 2-methylbutyric (0.01 g), isovaleric (0.01 g), valeric (0.05 g), and hexanoic acids (0.01 g) were dissolved in 100 mL water using a volumetric flask. An internal standard (IS) mixed solution of acetic-1-_13_C(C2) (12.21 mg/L), propionic-d_2_(C3) (2.28 mg/L), butryric-1-_13_C(C4) (1.35 mg/L), valeric-1-_13_C(C5) (0.10 mg/L), and hexanoic acid-d_11_(C6) (0.13 mg/L) was prepared. All stock solutions were stored in safe conditions at 4 °C and diluted with the salt-buffer solution before use.

Salting out was performed using NaCl and NaH_2_PO_4_ prepared at a 1.5 g/5 mL concentration, and the pH was adjusted to 2.0, 2.5, 3.0, and 3.5 using sulfuric acid and measured using a pH meter (Seven Compact S220, Mettler Toledo, Greifensee, Switzerland). For the fecal matrix effect, homogenized fecal samples (300 mg) were analyzed in 20 mL glass SPME vials (Gerstel, Mülheim an der Ruhr, Germany) containing the salt-buffer solution (5 mL). Approximately 100 mg of frozen fecal samples from different collection times was transferred into SPME vials, 5 mL of aqueous NaH_2_PO_4_ solution (pH 2.0) was added, and 10 µL of IS mix solution was added and immediately mixed.

### Animal and fecal sample collection

Seven-week-old male C57BL/6 J mice were obtained from Orient Bio (Gyeonggi-do, South Korea), housed in an SPF animal facility, and supplied with food and water ad libitum under controlled environmental conditions at 21 ± 2 °C in a light–dark room. The mice were fed a Teklad Global 18% Protein Rodent Diet (2918C; Harlan Teklad, Madison, WI, USA). All animal experiments were reviewed and approved by the Animal Welfare Committee of the Korea Food Research Institute (KFRI-M-22038). After a 3-week acclimation, fecal samples were collected immediately in a metabolic cage and after 4 and 12 h of cage change and immediately stored at − 80 °C until further use.

### Extraction using headspace SPME and GC–MS/MS analysis

Three types of fibers were tested to evaluate the adsorption of SCFAs: 23 Ga fiber coated with 85 μm CAR/PDMS, 50/30 μm DVB/CAR/PDMS, and 100 μm PDMS (Supelco, Bellefonte, PA, USA). Prior to their use, all fibers were heated in the thermal port of a multipurpose sampler (MPS; Gerstel, Sursee, Switzerland) according to the manufacturer’s recommendations. Next, compounds were extracted using the MPS with automated SPME sampling. The vial was heated and incubated at 60 °C for 10 min, with agitation on and off for 10 and 1 s at 300 rpm. The 85-μm CAR/PDMS fibers were exposed to the sample at 60 °C for 15 min after pre-bake out at 280 °C for 3 min. The extracted volatiles were desorbed using a GC injector at 250 °C for 1 min. To prevent analyte carryover, the SPME fibers were post-baked for 12 min after each extraction.

The SCFA were separated using a 7890A GC coupled with a 7000C TQ MS/MS (Agilent, Santa Clara, CA, USA) in a DB WAXetr capillary column of 30 m × 250 µm i.d., and 0.25 µm film thickness (Agilent). The initial temperature of the oven was set at 80 °C for 2 min, increased to 100 at 10 °C/min, ramped to 130 °C at the rate of 5 °C/min, followed by 160 °C at the rate of 10 °C/min and 220 °C at the rate of 20 °C/min, and finally held 2 min. A final temperature holds of 240 °C for 4 min was used to clean the column. Injections were performed in the splitless mode, and helium was used as the carrier gas at a constant flow of 1 mL/min. The total run time was 16 min with a solvent delay of 8 min.

The MS transfer line temperature was set at 240 °C, and the EI source and quad were kept at 230 °C and 150 °C. The triple quadrupole was operated with N_2_ as the collision gas at a flow of 1.5 mL/min and He as the quenching gas at a 2.25 mL flow. The multiple reaction monitoring (MRM) specifications for each compound are listed in supplementary Table 1.

### Data analysis

Relative and absolute quantification of SCFAs was performed using the MassHunter workstation software version B.07.00 (Agilent). Peak picking, peak area calculation, standard curve construction, limit of detection (LOD), and limit of quantification (LOQ) were conducted with signal-to-noise ratios of 3.3 and 10 for the diluted standard solutions, respectively. Each SCFA’s linearity was evaluated by calibration curves of internal standard, and linear regression analysis was generated to determine the slope, correlation coefficient, and intercept for each calibration curve. LOD and LOQ were calculated using the standard deviation of the regression line’s *y*-intercepts(r) and the slope(s). The respective formulas used were LOD = 3.3r/S and LOQ = 10r/S, respectively. The SCFA concentration in feces was quantified using a standard curve constructed based on the peak area adjusted using internal standards with the equivalent number of carbons for each SCFA.

Data are presented as mean ± standard deviation (S.D.). Extraction efficiency was determined based on the relative peak areas of the eight SCFAs. The precision and reproducibility of the method were evaluated using the coefficient of variation (CV), the ratio of S.D. to the mean, and expressed as percentages. Univariate analyses, such as the *T*-test, one-way analysis of variance (ANOVA), and Tukey’s multiple comparison range test, were conducted using the Prism software version 9 (GraphPad, San Diego, CA, USA).

## Results

### Performance of SPME fibers

**Fig. 1:**
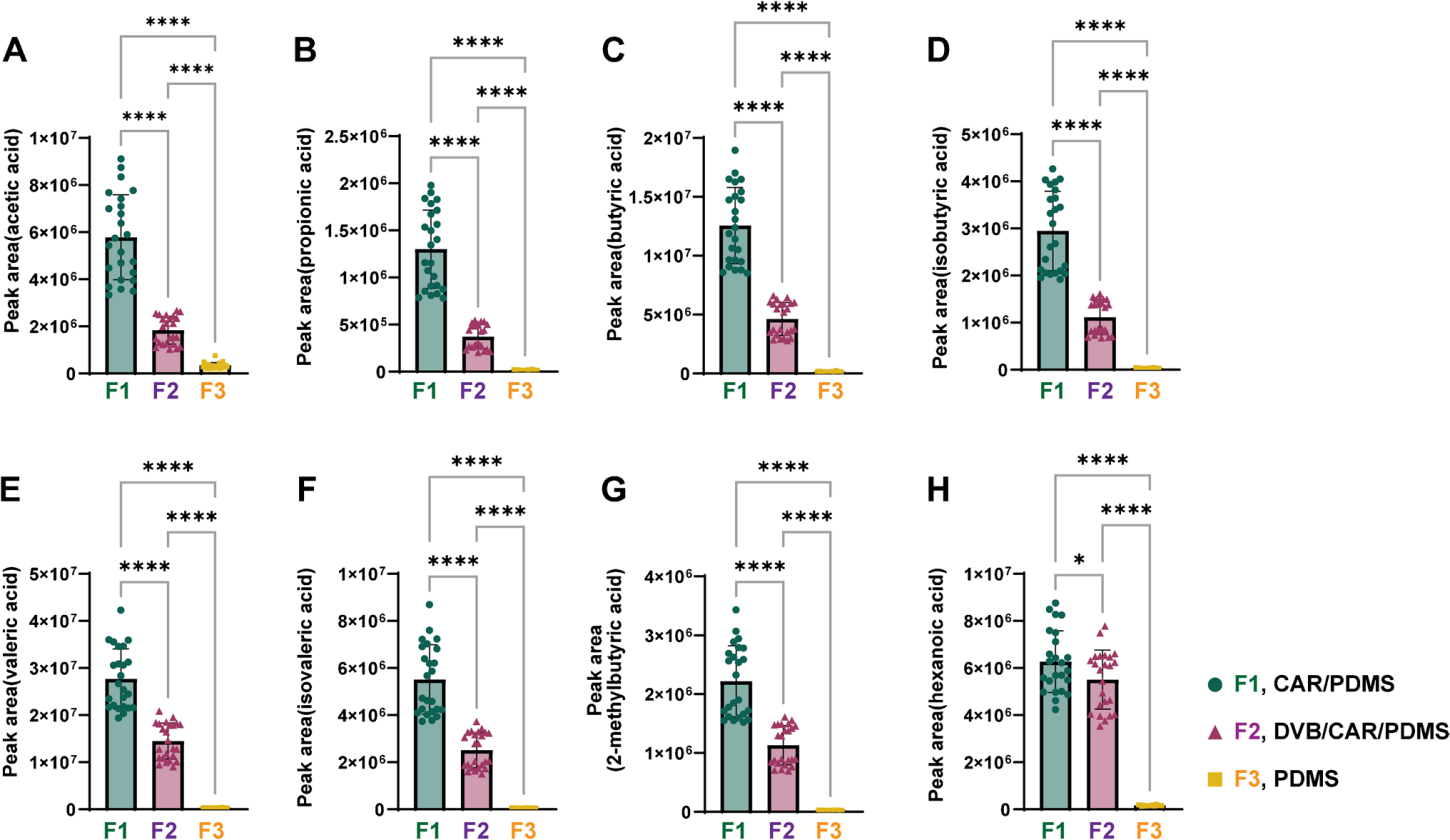
Effect of solid-phase microextraction (SPME) fibers on the extraction efficiency of eight short-chain fatty acids (SCFAs). Each panel represents the peak area of **A**: acetic acid, **B**: propionic acid, **C**: butyric acid, **D**: isobutyric acid, **E**: valeric acid, **F**: isovaleric acid, **G**: 2-methylbytyric acid and H: hexanoic acid. Three different types of fibers are presented as F1 (CAR/PDMS, green circle), F2 (DVB/CAR/PDMS, purple triangle), and F3 (PDMS, yellow square). The asterisks indicate significant difference (*p < 0.05, ****p < 0.0001)

We evaluated the SCFA extraction efficiency and reproducibility of the three types of SPME fibers by comparing their relative areas. Figure 1 demonstrates that the CAR/PDMS fiber (8.5 × 10^5^‒3.8 × 10^7^) had a significantly highest extraction efficiency of SCFAs, followed by the DVB/CAR/PDMS fiber (2.1 × 10^5^‒2.0 × 10^7^) and PDMS fiber (1.1 × 10^4^‒4.6 × 10^5^) (*p* < 0.05). The extraction efficiencies of the DVB/CAR/PDMS and PDMS fibers were 24.8‒57.9% and 0.7‒7.9%, respectively, compared to the CAR/PDMS fiber. Meanwhile, the coefficient of variation (CV%) value was the lowest in the CAR/PDMS fiber (1.2‒21.8%) compared to DVB/CAR/PDMS (0.5‒23.3%) and PDMS fibers (0.3‒58.4%), indicating that the CAR/PDMS fiber has high reproducibility. Due to its high extraction efficiency and reproducibility, CAR/PDMS fiber was selected for SCFA extraction and used in subsequent experiments to determine the salting-out effect of aqueous salt solutions.

### Optimization of pH for SCFA analysis

**Fig. 2:**
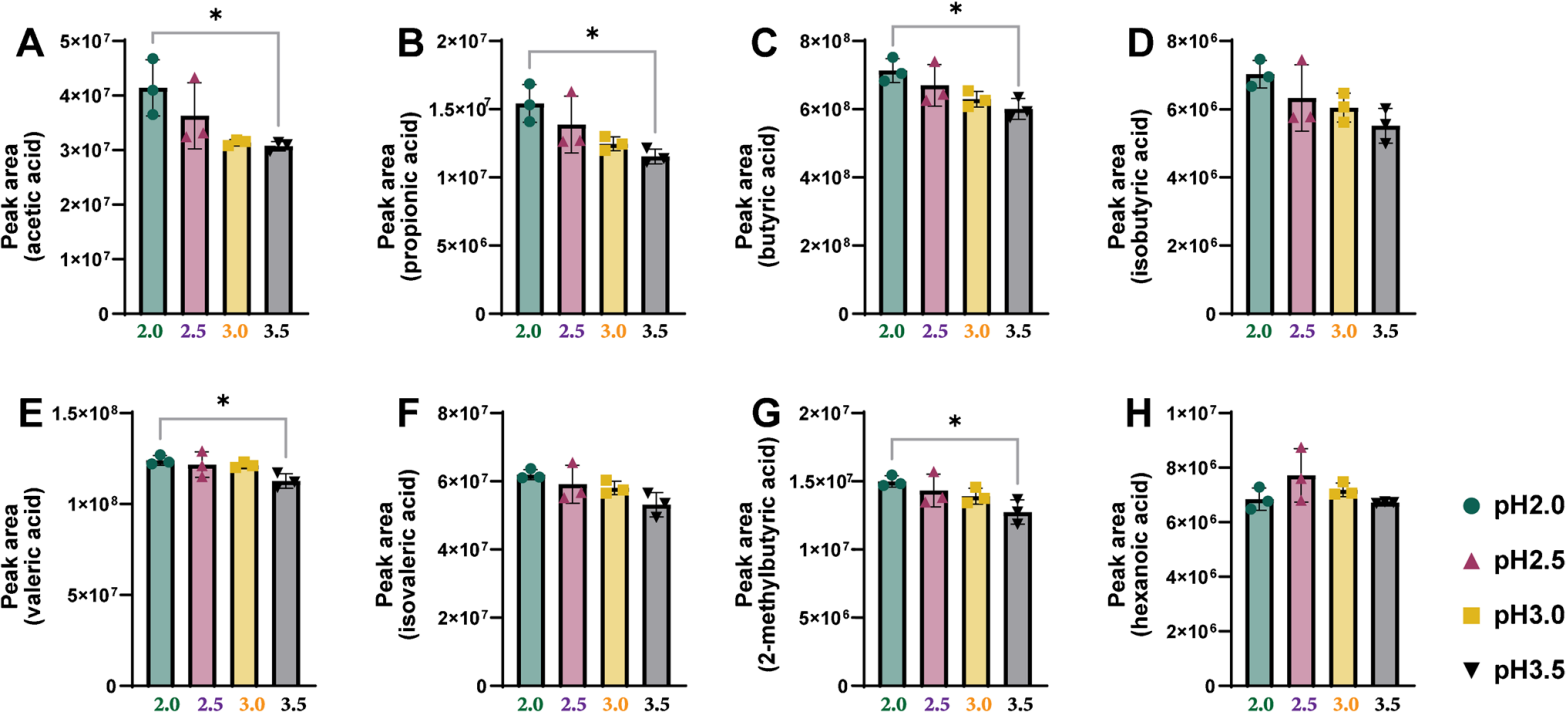
Effect of pH on the extraction efficiency of eight SCFAs. Each panel represents the peak area of **A**: acetic acid, **B**: propionic acid, **C**: butyric acid, **D**: isobutyric acid, **E**: valeric acid, **F**: isovaleric acid, **G**: 2-methylbytyric acid and H: hexanoic acid. The asterisks indicate significant difference (*p < 0.05)

To investigate the effect of pH on SCFA extraction, we analyzed the SCFAs present in mouse feces using aqueous NaH_2_PO_4_ solutions with different pH values. Figure 2 shows the effect of pH on the extraction efficiency of the eight mice fecal SCFAs. The relative area of SCFAs calibrated by fecal weight decreased as the pH increased, except for hexanoic acid, which showed a high level at pH 3.0. Acetic, propionic, butyric, valeric, and 2-methyl butyric acids showed significantly decreased values between pH 2.0 and 3.5 (*p* < 0.05). Based on these results, we optimized the pH of the aqueous NaH_2_PO_4_ solution to 2.0, which showed the most pronounced salting-out effect in the fecal samples.

### Salting-out effect in fecal SCFA analysis

**Fig. 3:**
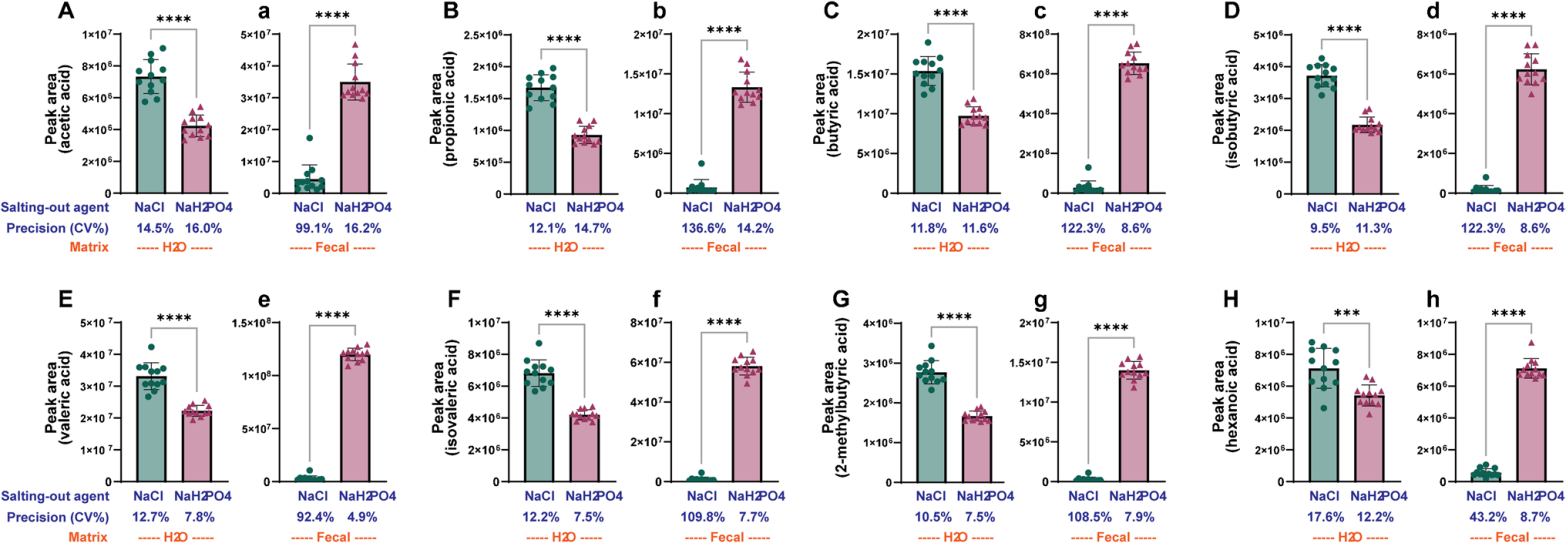
Salting-out effect of NaCl and NaH_2_PO_4_ and fecal matrix effect on the extraction efficiency of the eight SCFAs. Each panel represents the peak area of **A**: acetic acid, **B**: propionic acid, **C**: butyric acid, **D**: isobutyric acid, **E**: valeric acid, **F**: isovaleric acid, **G**: 2-methylbytyric acid and H: hexanoic acid. Capital and small letters represent the water and fecal matrix effect, respectively. The asterisks indicate significant difference (***p < 0.001, ****p < 0.0001)

We examined the amount of SCFA extracted using NaCl and NaH_2_PO_4_, which are the most commonly used salting-out agents in SPME analysis systems, to investigate their effect on SCFA extraction from mouse feces. The extraction efficiency of salting-out agents was evaluated according to the relative area of compounds. As shown in Fig. 3A–H, NaCl may be an effective salting-out agent for extracting all eight SCFAs from the water matrix. Meanwhile, in the mouse fecal matrix, NaH_2_PO_4_ was found to be a more effective agent, and the difference between the two salt agents was greater in the mouse fecal matrix. In the fecal matrix, the CV% value of NaCl ranged from 43.2 to 136.6%, whereas that of NaH_2_PO_4_ was only 4.9 to 16.2% (Fig. 3a‒h), indicating NaH_2_PO_4_ significantly decreases the CV% value compared to using NaCl. This interesting observation suggests that NaH_2_PO_4_ improves the reproducibility and reliability of SCFA analytical data. To examine this notable result, the pH of each aqueous salt solution was analyzed after adding mouse fecal samples, and the results are presented in Table 1. The pH of the aqueous NaCl solution rapidly increased from acidic conditions (pH 2.0–3.5) to neutral conditions (pH 6.5–6.8), while that of NaH_2_PO_4_ aqueous solution was very stable after adding mouse feces. Based on these observations, NaH_2_PO_4_ was selected as the salting-out agent for the SCFA analysis.

**Table 1:** The pH of the aqueous salts solution after adding mouse fecal sample

Salting-out agent (NaCl)			Salting-out agent (NaH_2_PO_4_)	
Initial pH	pH after adding feces		Initial pH	pH after adding feces
2.0	6.47 ± 0.06		2.0	1.96 ± 0.06
2.5	6.78 ± 0.04		2.5	2.42 ± 0.01
3.0	6.84 ± 0.05		3.0	3.09 ± 0.02
3.5	6.83 ± 0.05		3.5	3.55 ± 0.02

Data are mean ± S.D. (*n* = 3)

### Effect of sample collection time on SCFA analysis

**Fig. 4:**
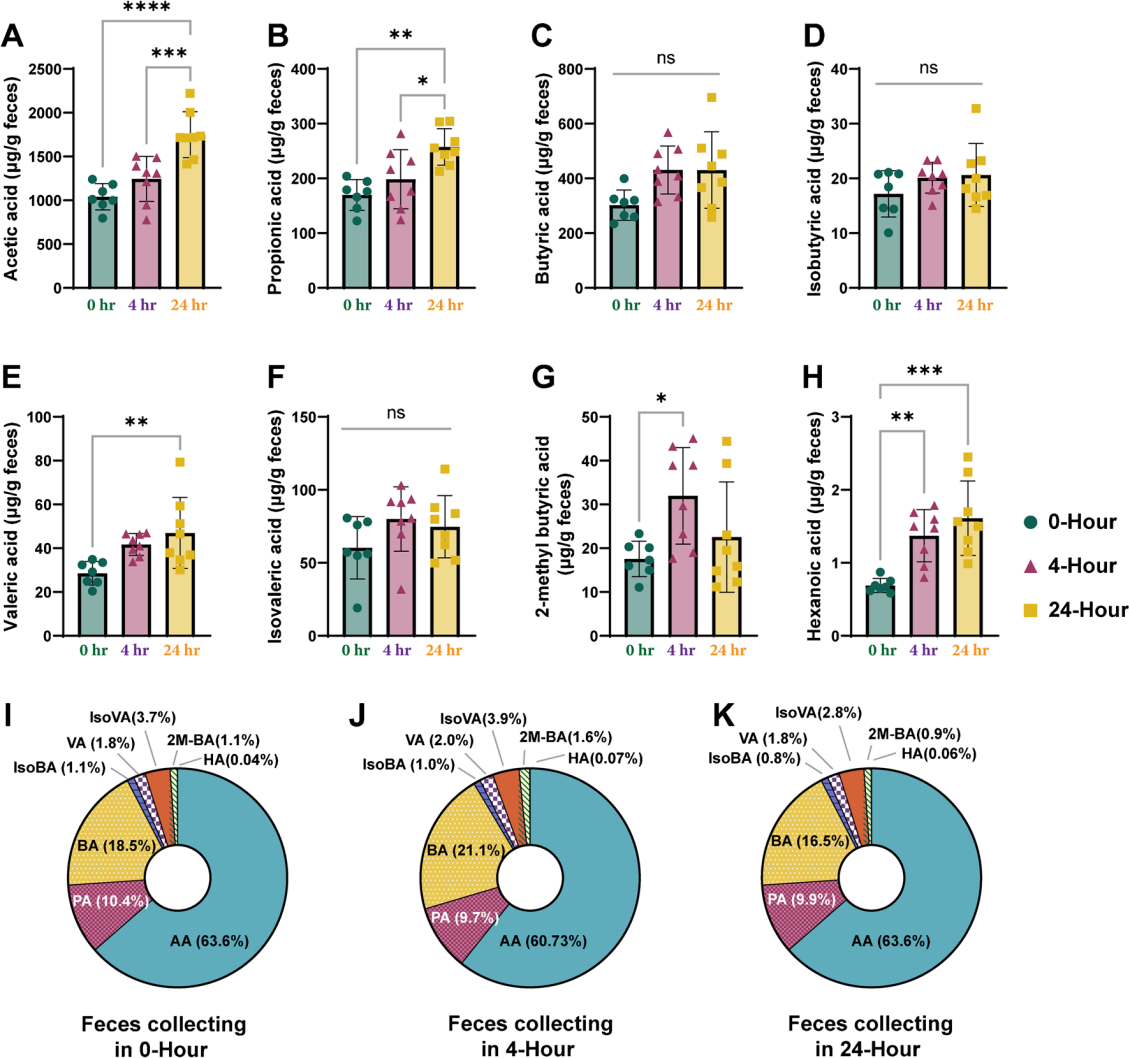
Effect of mouse fecal sample freshness in the analysis of eight SCFAs. Each panel represents the concentration of **A**: acetic acid, **B**: propionic acid, **C**: butyric acid, **D**: isobutyric acid, **E**: valeric acid, **F**: isovaleric acid, **G**: 2-methylbytyric acid and H: hexanoic acid. The peak area was divided by the internal standard peak area with the equivalent number of carbons for each SCFA. The asterisks indicate significant difference (*p < 0.05, **p < 0.01, ***p < 0.001, ****p < 0.0001)

We investigated the effect of the freshness of mouse fecal samples, which was based on the collection time, on the composition and concentration of SCFAs. We quantified eight SCFAs present in mouse fecal samples collected at three different time points using previously confirmed parameters, including the CAR/PDMS SPME fiber and an aqueous NaH_2_PO_4_ solution with a pH of 2.0 (Fig. 4). Results showed that the concentrations of SCFAs increased with later sample collection times, except for 2-methylbutanoic acid, which was found to be significantly higher in the 4-h sample collection (*p* < 0.05) (Fig. 4A‒H). In the overall proportion of SCFAs, the content of acetic, propionic, and butyric acids was the most abundant. Interestingly, as the fecal collection time was delayed, acetic and propionic acid concentrations, which constitute three-quarters of the total SCFA content, increased. This was due to an increase in CV%, which subsequently reduced the reliability of the SCFA analysis results. The SCFA composition was as follows: acetic acid (60.7‒67.2%) > butyric acid (16.5‒21.0%) > propionic acid (9.7‒10.4%) > isovaleric acid (2.9‒3.9%) > valeric acid (1.7‒2.0%) > 2-methylbutanoic acid (0.9‒1.6%) > isobutanoic acid (0.8‒1.0%) > hexanoic acid (0.04‒0.07%). Although the proportion of hexanoic acid significantly increased from 0.04% at 0 h to 0.07% at 4 h, this change did not affect SCFA composition (*p* < 0.05). Based on these results, we suggest that mouse fecal samples should be collected within 4 h to obtain reliable analytical results.

### LOD and LOQ of SCFAs using SPME–GC–MS/MS

The LOD and LOQ of the eight short-chain fatty acids are presented in Table 2. The LOQ and LOD of acetic acid were significantly higher than those of the seven other SCFAs. A calibration curve with six to eight points was plotted, and good linearity was obtained for all SCFAs: 3.063–404.840 μg for acetic acid, 0.286–38.200 μg for propionic acid, 0.149–19.920 μg for butyric acid, 0.145–19.400 μg for isobutyric acid, 0.155–20.760 μg for valeric acid, 0.028–3.840 μg for isovaleric acid, 0.037–4.920 μg for 2-methyl butyric acid, and 0.029–3.920 μg for hexanoic acid. All SCFAs had correlation coefficients greater than 0.999 of *R*-squared, except acetic acid (*R*^2^ = 0.9975). The LOD and LOQ values refer to the range used for analyzing SCFAs in mouse fecal samples using approximately 2–3 lumps or 50–70 mg of feces for analysis.

**Table 2:** The limit of quantitation (LOQ) and limit of detection (LOD) of eight short-chain fatty acids by SPME–GC–MS/MS

	LOD	LOQ	Linearity range (ug)	Calibration equation (*R*-squared value)	*y*-intercepts
Acetic acid	40.03	121.30	3.036–404.840	*Y* = 44,060*X* + 769,506 (*R*^2^ = 0.9975)	534,427.4
Propionic acid	0.353	1.071	0.286–38.200	*Y* = 1.8e + 5*X* − 56,863 (*R*^2^ = 0.9999)	19,249.9
Butyric acid	0.484	1.465	0.149–19.920	*Y* = 3.1e + 5*X* + 530,137 (*R*^2^ = 0.9999)	449,021.2
Isobutyric acid	1.306	3.957	0.145–19.400	*Y* = 7.1e + 5*X* + 139,439 (*R*^2^ = 0.9998)	281,380.1
Valeric acid	0.261	0.791	0.155–20.760	*Y* = 6.7e + 6*X* + 353,110 (*R*^2^ = 0.9999)	530,071.6
Isovaleric acid	0.194	0.589	0.028–3.840	*Y* = 7.0e + 6*X* + 72,301 (*R*^2^ = 0.9990)	413,765.8
2-methyl butyric acid	0.224	0.678	0.037–4.920	*Y* = 2.3e + 6*X* + 39,264 (*R*^2^ = 0.9999)	156,333.5
Hexanoic acid	0.115	0.348	0.029–3.920	*Y* = 1.4e + 7*X* − 612,973 (*R*^2^ = 0.9997)	475,033.5

## Discussion

Herein, we present an efficient and reproducible method for quantifying eight SCFAs containing up to four carbon atoms using GC–MS/MS. Various extraction and analytical factors affect the analysis; thus, we have investigated the performance of three SPME fibers, the pH condition during SCFA extraction, the salting-out agent, and the effect of mouse fecal sample freshness.

Selecting the type of SPME fiber is one of the primary considerations in headspace SPME–GC–MS analysis, and carboxen and PDMS are the most commonly used coating materials in SPME fibers. Carboxens are typically selected for the analysis of low-molecular-weight compounds such as alcohols and volatile organic compounds, whereas PDMS is suitable for non-polar to moderately polar compounds, including fatty acids, alcohols, and other volatile organic compounds [12]. In the present study, we found that the CAR/PDMS-coated fiber was the most efficient for extracting the eight SCFAs, compared to the DVB/CAR/PDMS and PDMS fibers. This result is inconsistent with a previous study that used the DVB/CAR/PDMS fiber type without performing a comparative test [13]. According to the supplier, DVB/CAR/PDMS is suitable for low-molecular-weight (C3–C20) volatiles and semi-volatiles, and it seems to be quite suitable for SCFA extraction. Our results suggest that the DVB layer, which provides additional selectivity for polar compounds, was not very effective in the analysis. Although several studies have used DVB/CAR/PDMS fibers [5, 14], we strongly suggest that a more specific CAR/PDMS fiber is more efficient at targeting SCFAs with 2–4 carbon atoms.

The pH of the sample phase affects analyte ionization that influences the acid–base equilibria between the sample phase and SPME fiber coating, thereby affecting the extraction efficiency. Although some substances can be sensitive to changes in pH, which can lead to their conversion into other substances, adjusting and maintaining an appropriate pH can improve extraction efficiency and accuracy. The present study found that a lower solvent pH led to higher extraction efficiency, indicating that the partitioning equilibrium ratio between the SCFAs in the sample phase and SPME fiber increased at a low pH of 2.0. This result was particularly noticeable for low-molecular-weight SCFAs, such as acetic, propionic, and butyric acids. Hexanoic acid showed the highest extraction efficiency at a pH of 2.5, suggesting that the optimal solvent pH for each analytical substance should be considered.

The salting-out effect in SPME extraction refers to the reduction in the solubility of a solute in an aqueous solution due to the presence of a high concentration of electrolyte salt [15]. This salting-out effect is another crucial factor in determining the extraction efficiency and sensitivity of SPME analysis, as it determines the partition coefficient between the sample and SPME fiber based on the salt concentration in the matrix [16]. Hence, choosing a suitable salting-out agent should be carefully considered to enhance both the quantity of extracted substances and analytical sensitivity. In this study, we compared the SCFA extraction efficiencies of the two most commonly used salting-out agents: NaCl and NaH_2_PO_4_. NaH_2_PO_4_ showed a significantly higher extraction efficiency for all eight SCFAs, suggesting that it is a more effective agent than NaCl for SCFA analysis. This is consistent with the findings of a previous study wherein the extraction efficiency of (NH4)_2_SO_4_/NaH_2_PO_4_ mixture was four times higher than that of NaCl [15]. However, we also observed that when NaCl was used as the salting-out agent, the pH of the sample matrix significantly shifted from 2.0 to 6.5, whereas the pH remained stable at 2.0 in NaH_2_PO_4_. As previously mentioned, low pH is a crucial factor in enhancing the extraction efficiency of SCFAs, which implies that NaCl interferes with maintaining a low pH of the solvent matrix. Furthermore, we observed a remarkable reduction in the CV% value when using NaH_2_PO_4_, resulting in increased data reproducibility and reliability.

Table 2 shows that the LOD of the eight SCFAs ranged from 0.115 to 40.03 μg/g feces, and the LOQ ranged from 0.348 to 121.30 μg/g feces. Fu et al. [17] extracted 11 SCFAs in mouse feces by a combined method of SPME and chemical derivatization and analyzed by GC–MS/MS, and reported LOD and LOQ ranged from 0.01 to 0.72 ng/mL and 0.04 to 2.41 ng/mL, respectively. Meanwhile, Scortichini et al. [18] studied SCFAs in rat and human feces by GC-FID and reported LOD ranging from 0.04 to 0.64 μM and LOQ ranging from 0.14 to 2.12 μM. The LOD and LOQ differences likely originated from each study’s equipment, columns, and sample preparation method. Although the LOD and LOQ of this study are higher than those of other studies, they are low enough to quantify SCFAs in mouse fecal samples.

As interest in the microbiome has increased, research on the microbial diversity in human stool samples has been conducted, and appropriate protocols have been established [19, 20]. However, no studies have investigated the microbial or component changes in mouse fecal samples collected using varying methods and times. In numerous clinical studies, standardized methodologies on the procurement, preservation, and transportation of fecal specimens have been delineated [21, 22], such as the finding that sample transportation faster than two days did not affect the microbial community in a human stool sample. However, in terms of mouse fecal samples, the effects of collection time and method on the analytical results have not been reported yet. Although previous studies have not explicitly mentioned how rapidly mouse fecal samples are collected, most researchers try to collect samples as promptly as possible and immediately freeze them [23, 24]. Housing a mouse in a metabolic cage for as little as one hour to as much as three hours and collecting feces causes considerable stress not only to the researcher but also to the animal. This indicates that the fecal collection process for SCFA analysis may also interfere with the aim of the study. Therefore, we attempted to reduce stress in the experimental animals by analyzing the change in fecal SCFA content over time when the bedding of the animal cage was changed. According to the quantitative analysis, the SCFA concentrations increased depending on the sample collection time, except for 2-methylbutanoic acid. This was considered to be the result of evaporation and water concentration of the fecal sample in the mouse-rearing environment. In addition, as shown in Fig. 4I–K, the composition ratio of the eight SCFAs did not change significantly according to the collection time. This result may be due to the fact that all eight fecal SCFA are not easily volatilized in the mouse breeding environment. We conclude that sample collection within four hours is reasonable for obtaining reliable results in mouse fecal SCFA analysis. Researchers do not have to collect mouse fecal samples immediately after excretion, thereby reducing the stress in the mouse-rearing environment and the effort of researchers. In this study, we implemented various strategies to enhance extraction efficiency, aiming to overcome the limitation of an extremely small amount of sample when conducting quantitative analysis of SCFAs from mouse feces samples. This experimental method developed in this study will provide a systematic approach for the quantitative analysis of SCFAs in limited quantities of mouse fecal samples. Additionally, it is considered that further research will be required to examine the extraction efficiency of volatile low-molecular compounds in fecal samples over the eight types of SCFAs analyzed in this study.

## Conclusion

This study aimed to optimize a method for quantifying eight SCFAs using triple quadrupole GC–MS/MS and presented the variables that could affect the analysis results. Results showed that the CAR/PDMS-coated SPME fiber was the most efficient for extracting SCFAs, contrary to past research that favored DVB/CAR/PDMS fibers without comparative testing. The pH of the sample phase significantly affected extraction efficiency, with a pH of 2.0 leading to better results. This was particularly evident for low-molecular-weight SCFAs, suggesting that an optimal solvent pH should be considered for each substance. Compare to NaCl, NaH_2_PO_4_ was found as a more effective salting-out agent in extracting SCFAs because of its stable pH after feces addition, whereas NaCl interferes with maintaining the solvent matrix’s low pH. The study also analyzed the impact of collection time on SCFA composition and concentration, and the results showed that SCFA concentrations increased over time because of moisture evaporation. However, the composition ratio of the eight SCFAs did not significantly change with time; thus, we concluded that samples should be collected within four hours to obtain reliable results.

### Supplementary information

Below is the link to the electronic supplementary material.

Supplementary file1 (DOCX 138 KB)
