# Supplementary material for: Analytical factors for eight short-chain fatty acid analyses in mouse feces through headspace solid-phase microextraction–triple quadrupole gas chromatography tandem mass spectrometry
Source: Anal Bioanal Chem. 2023 Aug 17;415(25):6227–35. doi: 10.1007/s00216-023-04895-1 (PMC10558374; doi:10.1007/s00216-023-04895-1)
Supplement: Supplementary file 1 — Supplementary file1 (DOCX 138 KB) [file 216_2023_4895_MOESM1_ESM.docx]

**[Supplementary results]**

**Table S1. Multiple Reaction Mode (MRM) transitions used for SCFAs by GC-MS/MS**

| Compound | | RT  (min) | DT (ms) | ion pairs* | CE  (ev) | ion pairs** | CE  (ev) | ion pairs** | CE  (ev) |
| --- | --- | --- | --- | --- | --- | --- | --- | --- | --- |
| C2 | Acetic acid | 8.17 | 10 | 60>43 | 5 | 60>45 | 10 | - |  |
|  | Aectic acid-1-^13^C  (IS) | 8.17 | 10 | 61>44 | 5 | 61>46 | 10 | - |  |
| C3 | Propionic acid | 9.85 | 8 | 74>73 | 5 | 74>46 | 10 | 74>55 | 10 |
|  | Propionic acid-d_2_  (IS) | 9.80 | 8 | 76>75 | 1 | 76>57 | 16 | - |  |
| C4 | Isobutyric acid | 10.41 | 15 | 73>55 | 5 | 88>73 | 5 | 88>55 | 10 |
|  | Butyric acid | 11.50 | 10 | 60>43 | 10 | 73>55 | 5 | - |  |
|  | Butyric acid-1-^13^C  (IS) | 11.50 | 10 | 61>43 | 10 | 74>56 | 10 | - |  |
| C5 | 2-methylbutyric acid | 12.17 | 10 | 74>73 | 5 | 74>56 | 5 | - |  |
|  | Isovaleric acid | 12.18 | 10 | 60>42 | 10 | 60>45 | 10 | - |  |
|  | Valeric acid | 13.21 | 9 | 60>42 | 10 | 60>45 | 10 | 73>55 | 5 |
|  | Valeric acid-1-^13^C  (IS) | 13.21 | 9 | 67>43 | 12 | 74>56 | 8 | - |  |
| C6 | Haxanoic acid | 14.45 | 9 | 60>42 | 10 | 60>45 | 10 | 73>55 | 5 |
|  | Hexanoic acid-d_11_  (IS) | 14.31 | 9 | 63>44 | 10 | 77>58 | 10 | - |  |

^*^Quantitative ion, ^**^Qualitative ion.

CE, collision energy; DT, dwell time; IS, internal standard; RT, retention time.

**
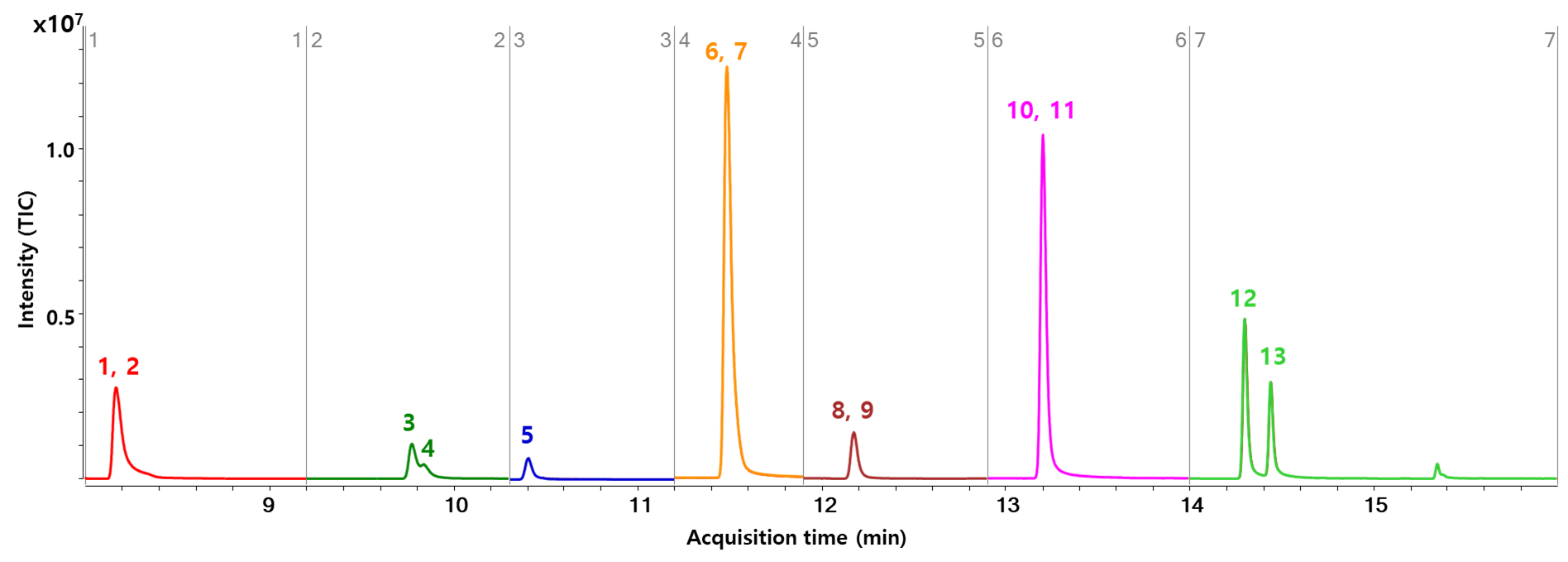
**

Fig S1. GC-MS/MS chromatogram of SCFA of standard mixture, 1, Acetic acid; 2, Aectic acid-1-^13^C; 3, Propionic acid-d_2_; 4, Propionic acid; 5, Isobutyric acid; 6, Butyric acid; 7, Butyric acid-1-^13^C; 8, 2-methylbutyric acid; 9, Isovaleric acid; 10, Valeric acid; 11, Valeric acid-1-^13^C; 12, Hexanoic acid-d_11_; 13, Haxanoic acid.
